# Anti-inflammatory effects of ivy leaves dry extract: influence on transcriptional activity of NFκB

**DOI:** 10.1007/s10787-018-0494-9

**Published:** 2018-05-11

**Authors:** Janka Schulte-Michels, Christina Keksel, Hanns Häberlein, Sebastian Franken

**Affiliations:** Institute of Biochemistry and Molecular Biology, Rheinische Friedrich-Wilhelms-University, Nussallee 11, 53115 Bonn, Germany

**Keywords:** NFκB, Hedera helix, β_2_-Adrenergic receptor, Airway inflammation, EA 575^®^

## Abstract

EA 575^®^ is an ivy leaves dry extract (DER 5-7.5:1, 30% ethanol) used against diseases of the lower respiratory tract associated with productive cough. EA 575^®^ improves symptoms associated with chronic inflammatory bronchial conditions. Compared to its bronchospasmolytic and secretolytic properties, the anti-inflammatory effects of EA 575^®^ are mostly untried. Therefore, we addressed the question of whether the anti-inflammatory effect of EA 575^®^ is due to an impact on the NFκB pathway. NFκB nuclear translocation was visualized by immunofluorescence in J774.2 as well as HEK293 cells. In the latter, a luciferase-based reporter was used to monitor NFκB transcriptional activity. Phosphorylation of RelA and its inhibitor IκB was measured by Western blot analysis. Additionally, changes in the stability of NFκB:IκB complex were shown by protein fragment complementation. Decreased transcriptional activity of NFκB under treatment with EA 575^®^ was also shown for a human monocytic as well as a human lung epithelial cell line. EA 575^®^ is able to inhibit NFκB transcriptional activity by partially inhibiting its translocation to the nucleus after stimulation with TNFα. Furthermore, phosphorylation of IκBα is reduced while phosphorylation of RelA is enhanced after pre-incubation with EA 575^®^, leading to an enhanced stability of NFκB:IκBα complex. EA 575^®^ has an regulatory impact on the NFκB pathway, possibly by switching specificity of IKK from IκBα to RelA, resulting in enhanced stability of NFκB:IκBα complex and reduced RelA translocation into the nucleus.

## Introduction

EA 575^®^ is an ivy leaves dry extract (DER 5-7.5:1, 30% ethanol) used for the improvement of symptoms associated with chronic inflammatory bronchial conditions and acute respiratory tract inflammation accompanied by coughing. Its bronchospasmolytic and secretolytic effects have been proven in clinical studies (Lang et al. 2015). The mechanism of action is based on increased β_2_-adrenergic responsiveness of the respiratory tract mediated by inhibition of GRK2-mediated phosphorylation of activated β_2_-adrenergic receptors, resulting in decreased internalization of β_2_-adrenergic receptors under stimulating conditions (Sieben et al. 2009; Schulte-Michels et al. 2016b). The anti-inflammatory effect of EA 575^®^ is less examined. As we have shown previously, EA 575^®^ is able to reduce LPS-induced IL-6 release from murine macrophages (J774.2) (Schulte-Michels et al. 2016a). Besides the findings from cell culture experiments, there is also growing evidence from animal models regarding the anti-inflammatory effect of constituents of hedera helix leaves. Rutin and chlorogenic acid reduce the carrageenan-induced paw edema in rats (Selloum et al. 2003; dos Santos et al. 2006). Rutin also inhibits acute as well as chronic arthritis states in rats (Guardia et al. 2001). Through a mouse model of ovalbumin-induced allergic asthma, it was demonstrated that chlorogenic acid reduces the synthesis of IgE and Th2-cell specific cytokines like IL-4 and IL-5 (Kim et al. 2010). Additionally, chlorogenic acid protects mice against LPS-induced lung injury (Zhang et al. 2010).

Hocaoglu et al. (2012) demonstrated that an ivy leaves dry extract showed anti-inflammatory properties in a chronic-induced asthma model in mice (Hocaoglu et al. 2012). Compared to placebo, goblet cell count was significantly lower and basal membrane thickness was reduced in lung histopathologic studies when mice were treated orally with the ivy leaves dry extract. Süleyman and coworkers also found an anti-inflammatory effect for an ivy extract enriched with saponins in cotton pellet and carrageenan-induced acute and chronic inflammation in rats (Süleyman et al. 2003).

An important aspect of nearly all inflammatory processes is the transcription factor NFκB. Under non-stimulating conditions, NFκB is kept inactive in the cytosol by complexation with its inhibitor IκBα. Stimulation of, e.g., macrophages with TNFα or LPS activates the IκB kinase (IKK), which in turn phosphorylates IκBα, resulting in a cleavage of the NFκB:IκBα complex. The released NFκB can thus be translocated into the nucleus, where it induces the transcription of different cytokines (Hayden and Ghosh 2012). Due to its relevance to inflammatory processes, we questioned if the reduced IL-6 release from EA 575^®^ pre-treated murine macrophages could be caused by an impact on the NFκB pathway.

In this work, we describe the influence of EA 575^®^ on the transcriptional activity of NFκB in different epithelial and immune system-derived cell lines using a Nanoluc reporter cell system. The nuclear translocation of NFκB after TNFα-stimulation is examined by immunofluorescence microscopy; the phosphorylation of IκBα and the stability of the NFκB:IκBα complex is investigated using Western blots and a protein fragment complementation assay, respectively. We aim to elucidate the anti-inflammatory mode of action of ivy leaves dry extract EA575^®^, and to further understand its beneficial effect on chronic inflammatory bronchial conditions.

## Materials and methods

### Chemicals

Nano-Glo^®^ Luciferase Assay was obtained from Promega (Promega GmbH, Mannheim). Ivy leaves dry extract EA 575^®^ (CID-100048) was obtained from Engelhard (Engelhard Arzneimittel GmbH & Co. KG Niederdorfelden).

The reference substances rutin, hederacoside C, hederacoside D, and α-hederin were obtained from Phytolab (Vestenbergsgreuth, Germany).

All other reagents were obtained from Merck (Darmstadt, Germany), if not stated otherwise.

### Characterization of EA 575^®^ by HPLC analysis

Ivy leaves dry extract EA 575^®^ (DER 5-7.5:1, 30% ethanol) (Batch number CID-100048) was obtained from Engelhard Arzneimittel GmbH & Co. KG (Niederdorfelden, Germany). Chemical composition and pharmacological properties of relevant ingredients have been reported previously (Sieben et al. 2009; Greunke et al. 2015; Schulte-Michels et al. 2016a, b). For further characterization, EA 575^®^ was dissolved in 50% EtOH in a concentration of 40.8 mg/ml. EA 575^®^ was analyzed on a Agilent Series 1200 HPLC system equipped with a degasser (G1322A), a quaternary pump (G1311A), an autosampler (G1329A) and a photodiode array detector (G1315D) using a LiChrospher RP-18 column (5 µm, 125 × 4 mm, Merck, Darmstadt). Solvent A was H_2_O/acetonitrile (44/2, m/m) adjusted to pH 2.0 with phosphoric acid 85%. Solvent B was acetonitrile. The following linear gradient was used: 0–9 min 0% B, 9–10 min to 6% B, 10–25 min to 15% B, 25–50 min to 60% B, 50–51 min to 100% B, 51–65 min 100% B. Flow rate: 0–50, 51–65 min 1.5 ml/min. Detection: 205 nm. Identification of constituents was carried out by comparison of UV spectra of reference substances and their corresponding retention times. Chromatograms were registered and evaluated using Agilent Chemstation Software Version B.04.01.

### Cell culture

The mouse macrophage cell line J774.2 was obtained from Sigma-Aldrich (Cat. Nr.: 85011428). J774.2 cells were cultured in DMEM without phenol red (Life Technologies, Carlsbad, CA) supplemented with 100 units/ml penicillin, 100 µg/ml streptomycin, 2 mM l-Glutamin (Life Technologies) and 10% fetal calf serum (Life Technologies). Cells were maintained by ten-fold dilutions with fresh medium every 2–3 days.

Human embryonic kidney cells (HEK 293) obtained from DSMZ (No. ACC 305) (Braunschweig, Germany) were cultivated in DMEM medium (Life Technologies, Carlsbad, CA) supplemented with 100 units/ml penicillin, 100 µg/ml streptomycin, and 10% fetal calf serum. Cells were maintained by threefold dilutions with fresh medium every 2–3 days.

Acute monocytic leukemia cells (THP-1) were obtained from DSMZ (No. ACC 16) and maintained in RPMI medium supplemented with 10% fetal calf serum, 100 units/ml penicillin, 100 µg/ml streptomycin, 50 µM β-mercaptoethanol and 5 ml Glutamax 100× (Life Technologies GmbH, Darmstadt). THP-1 cells were maintained by three-fold dilutions with fresh medium every 2–3 days.

Human lung epithelium-derived cells (A549) obtained from DSMZ (No. ACC 107) were cultivated in DMEM/F12 medium (Life Technologies, Carlsbad, CA) supplemented with 100 units/ml penicillin, 100 µg/ml streptomycin, and 10% fetal calf serum. Cells were maintained by three-fold dilutions with fresh medium every 2–3 days.

### Immunofluorescence

J774.2 or HEK293 cells were seeded on poly-d-lysine (PDL) coated coverslips in a density of 100,000 cells per well. At a cell density of 90% medium was changed to serum- and antibiotic-free medium. J774.2 cells were incubated with 80 µg/ml EA 575^®^ HEK293 cells with 240 µg/ml EA 575^®^ overnight. Serum-free medium served as a control. The next morning 100 ng/ml LPS and 25 ng/ml TNFα were added to J774.2 cells and HEK293 cells, respectively, and incubation was continued for another 50 min. Afterwards cells were fixed with 4% formaldehyde in PBS, permeabilized with ice-cold methanol and blocked with 5% BSA in PBS supplemented with 0.3% Triton X-100. Immunostaining was performed with RelA antibody (cell signaling technology, #6956) in a dilution of 1:800 overnight and a Cy3 coupled secondary antibody in a dilution of 1:400 for 1.5 h in the dark. Additionally, cells were incubated with Hoechst 33528 for nuclear staining. Images were taken on a Zeiss Axiovert 200 M with an LCI Plan-Neofluar 63×/1.30 Imm Korr Ph 3 M27 objective and an AxioCamMR.

### Generation of plasmids for protein fragment complementation

Generation of protein fragment complementation plasmids, pCDNAHygr_cVenus-RelA and pCDNA zeo_IκBa-nVenus, was already described elsewhere (Yu et al. 2003) and was constructed by GeneArt (Thermo Fisher), according to the mentioned publication.

### Generation of NFκB Reporter Plasmid

For the generation of a Nanoluciferase expression construct under control of NFκB binding sequence the pNF-KB-D2EGFP vector (Clontech) was used as a template. Destabilized GFP was removed from the vector by PCR introducing a new XhoI-site (forward primer: 5′TCGGATATCTCGAGCCGGAATTCGGGGAAGCTTC–3′; reverse primer: 5′–GTTCAGGGGGAGGTGTG–3′). The open reading frame coding for destabilized Nanoluciferase was cut from pNL1.2[*NlucP*] vector (Promega) using BamHI/XhoI and introduced into the pNF-KB vector using the same restriction sites.

### Transfection

HEK 293 cells were transfected by calcium phosphate method. Briefly, cells were seeded in 12 well plates and allowed to attach for at least 24 h. Before transfection medium was changed to 900 µl fresh medium. One µg DNA was mixed with 6.5 µl 2 M CaCl_2_ and 50 µl sterile water. The dilution was added dropwise to 2 × HBS and after half an hour of resting added to the cells. After 24 h, medium was changed to fresh DMEM containing antibiotic for selection (750 µg/ml geneticin).

THP-1 cells were transfected by Amaxa electroporation technology, Nucleofector^®^ II. For each transfection 2× 10^6^ cells were diluted in a self-made appropriate buffer (5 mM KCl, 15 mM MgCl_2_, 15 mM HEPES, 150 mM Na_2_HPO4/NaH_2_PO4 pH 7.2, 50 mM NaCl) with 1 µg DNA and transfected with program T-020. Afterwards, they were transferred to fresh fully supplemented RPMI medium. Twenty-four hours later, medium was changed to full medium containing 700 µg/ml geneticin for selection with medium changes every 2–3 days. After 1 week of selection, surviving cells were tested for expression of the transgene.

A549 cells were transfected using branched polyethylenimine (PEI, # 408727, Sigma-Aldrich, Darmstadt, Germany). Therefore, 50,000 cells were seeded into the cavities of a 12-well plate. 2 μg of DNA was diluted into 40 µl of 150 mM NaCl and 3.3 µl of a 1 mg/ml PEI solution was added and the mixture was immediately vortexed for 10 s. After 10 min at RT, the DNA/PEI mixture was applied dropwise to the cells and incubated overnight. The next day medium was changed to fresh DMEM/F12 containing antibiotic for selection (500 µg/ml geneticin).

### Measurement of NFκB activation through a Nanoluciferase-based reporter system

Nanoluciferase expressing cells were seeded in 96-well LumiNunc™ plates and were allowed to grow for 24 h. Medium was changed to serum-free medium before incubation. Incubation with EA 575^®^ was performed with indicated concentrations of EA 575^®^ for 8 h, respectively. After 5 h indicated concentrations of TNFα or LPS (THP-1 cells) were added for 3 h. Measurement of luciferase activity was performed using the Nano-Glo^®^ Luciferase Assay system (Promega, Mannheim, Germany) according to the manufacturers’ instructions.

### Western blot analysis

HEK 293 cells were seeded in 12-well plates and allowed to grow to a confluency of 80–90% in full-growth medium. Before incubation, the medium was changed to serum-free medium. Cells were incubated with 240 µg/ml EA 575^®^ for 8 h and with 25 ng/ml TNFα for 10 min. After the incubation, cells were harvested with 100 µl per well 10 mM HEPES buffer containing phosphatase inhibitor cocktail (Merck, Darmstadt, Germany) and protease inhibitor cocktail (Sigma-Aldrich, Darmstadt, Germany). Cell solutions were sonified for 15 s and lysed by being forced through a 30 G cannula. To separate cell debris, the solution was centrifuged at 15,000*g* for 5 min at 4 °C. Supernatant was diluted in 4× sample buffer and 20 µl of the samples was separated in a 10% acrylamide gel with 80 V in stacking and 120 V in resolving gel. Blotting was done with 200 mA per gel for 45 min. For normalization the membrane was stained after blotting with REVERT™ total protein stain according to the manufacturers’ instructions (LI-COR^®^). Intensity was measured on an LI-COR^®^ Odyssey reader in 700 nm channel. Afterwards, the stain was removed and membrane was blocked with Odyssey blocking buffer for 1 h. Immunostaining was performed using phospho-IκBa antibody (Cells Signaling Technology, #9246) or phospho-RelA antibody (Cell Signaling Technology, #3033), respectively, in a dilution of 1:1000 in Odyssey blocking buffer diluted 1:1 with TBS and 0.1% Tween 20. Afterwards, membrane was washed three times in TBS/Tween 20 and incubated in secondary antibody (LI-COR^®^ Goat anti Rabbit-DyLight 800, 1:10,000) diluted in Odyssey blocking buffer 1:1 with TBS, 0.1% Tween 20 and 0.01% SDS for 1 h. Membrane was washed three times in TBS and measured on an LI-COR^®^ Odyssey Reader in 800 nm channel. Protein concentration was normalized through the band intensity pIκBα/p-RelA divided by the normalization factor of REVERT™ total protein stain.

### Protein fragment complementation assay

HEK cells stably expressing IκBα-nVenus and cVenus-RelA were seeded into a black 96-well Plate (CellCarrier™, Perkin Elmer, Massachusetts, USA) in total growth medium and allowed to grow to a confluency of about 90%. After reaching confluency, medium was changed to HBSS buffer containing 20 mM HEPES and was pre-incubated with the indicated concentration of EA 575^®^ for 5 h. Afterwards, cells were stimulated with 10 ng/ml TNFα and decrease of fluorescence intensity was measured for 3 h every 60 min in a Perkin Elmer EnSight™ reader with excitation at 515 nm and emission at 550 nm. Additionally, cell confluency was measured in transmission channel. Data were registered and evaluated by Kaleido™ software.

### Statistical data evaluation

Statistical data evaluation was performed with One-way ANOVA followed by Tukey multiple comparison test. Results were considered to be significant for *p* values of at least < 0.05.

## Results

### EA 575^®^ analysis by HPLC

**Fig. 1:**
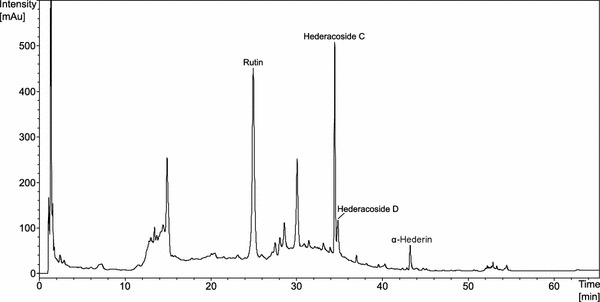
HPLC fingerprint analysis of EA 575^®^. Ingredients of the ivy leaves dry extract were separated on an RP-18 column. Main components were identified by comparison to retention times and absorbance of reference standards. The substances identified are rutin, hederacoside C, hederacoside D, and α-hederin

Composition of EA 575^®^ (DER 5-7.5:1, 30% ethanol) was characterized by HPLC fingerprint analysis. The following substances could be identified by comparison of UV spectra and retention times of corresponding reference substances: rutin, hederacoside C, hederacoside D, and α-hederin (Fig. 1).

### Partial inhibition of NFκB translocation into the nucleus

**Fig. 2:**
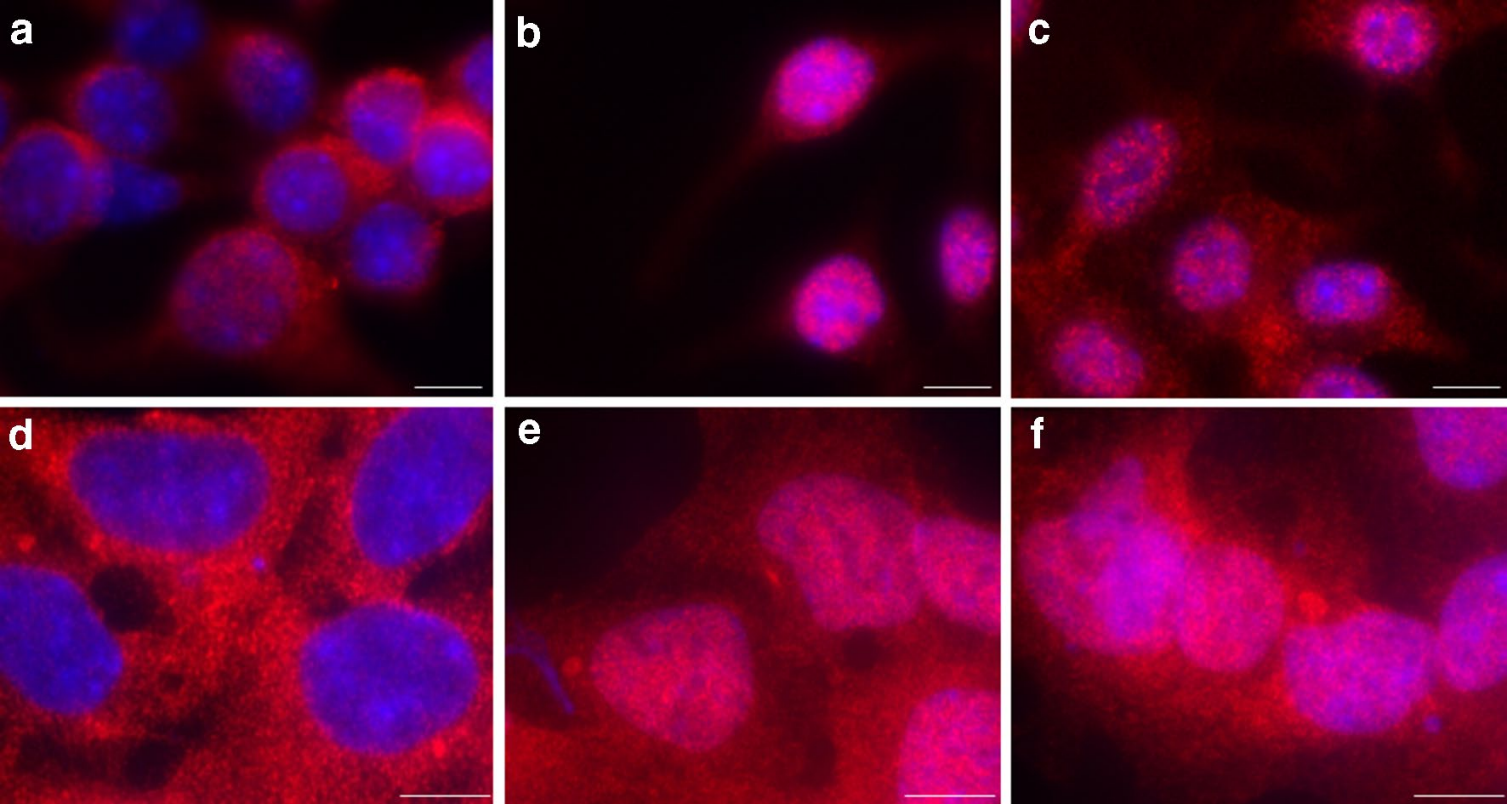
NFκB immunostained in red with anti-RelA primary antibody and Cy3 coupled secondary antibody shown in J774.2 (**a**–**c**) as well as HEK 293 cells (**d**–**f**). Nucleus stained in blue with Hoechst 33258. (**a** + **d**) unstimulated cells, (**b** + **e**) cells stimulated with 100 ng/ml LPS (J774.2) or 25 ng/ml TNFα (HEK293) for 50 min, and (**c** + **f**) cells pre-incubated with 80 µg/ml (J774.2) or 240 µg/ml EA 575^®^ (HEK293) overnight and subsequently stimulated with 100 ng/ml LPS (J774.2) or 25 ng/ml TNFα (HEK293) for 50 min. Scale bars correspond to 10 µm. Shown pictures are representative of 3 independent experiments

As previously shown, pre-incubation with EA 575^®^ reduces IL-6 secretion from murine macrophage cell line J774.2 (Schulte-Michels et al. 2016a). To examine whether this reduction is achieved by an inhibition of nuclear translocation of NFκB, we performed an immunofluorescence assay on NFκB subunit RelA in J774.2 cells. Incubation with 25 ng/ml TNFα for 50 min showed a considerable shift of NFκB (detected by Cy3 conjugated secondary antibody fluorescence) from cytosol to the nucleus (Fig. 2b) when compared to cells only incubated in serum-free medium (Fig. 2a). Overnight pre-incubation with 80 µg/ml EA 575^®^ observably reduced the signal located in the nucleus after 50 min of stimulation (Fig. 2c). In parallel, nuclear translocation of NFκB was examined in human embryonic kidney epithelial cells (HEK 293; Fig. 2d–f). Reduction of translocation was also detected in these cells, even though the effect was not as pronounced as in the macrophage cell line.

### Nanoluciferase based NFκB reporter assay

**Fig. 3:**
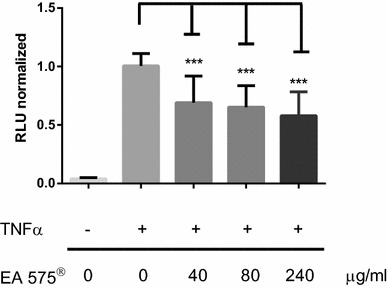
HEK 293 cells stably transfected with a Nanoluciferase based NFκB reporter plasmid were incubated for 8 h with the indicated amounts of EA 575^®^ or solvent alone. After 5 h of pre-incubation cells were stimulated for further 3 h with 25 ng/ml TNFα or left unstimulated. Results represent the mean ± SD (*n* ≥ 30, ***p < 0.001)

As shown previously by El-Guendy and colleagues, NFκB transcriptional activity can be measured by an NFκB reporter gene assay (El-Guendy and Sinai 2008). Stably transfected HEK 293 Nano-PEST cells showed an almost 25-fold increase in Nanoluciferase activity after stimulation with 25 ng/ml TNFα for 3 h. This increase could be significantly and dose-dependently decreased by 31.0, 34.7, and 42.1% after 8 h of pre-incubation with EA 575^®^ concentrations of 40, 80, and 240 µg/ml, respectively (Fig. 3).

### Western blot against phosphorylated IκBα and RelA

**Fig. 4:**
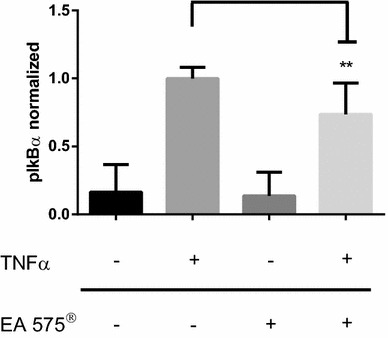
Phosphorylation of IκBα on Ser32 before and after stimulation with 25 ng/ml TNFα for 10 min and with or without pre-treatment with 240 µg/ml EA 575^®^ for 8 h. Results represent the mean ± SD (*n* ≥ 8, **p < 0.01)

**Fig. 5:**
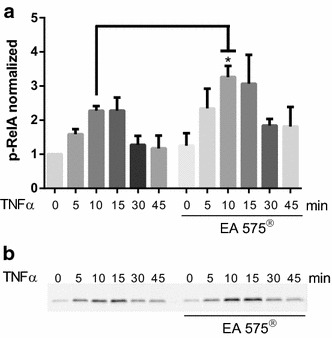
Phosphorylation of RelA on Ser536 (p-RelA) in HEK cells after stimulation with 25 ng/ml TNFα for different time periods with or without pre-treatment using 240 µg/ml EA 575^®^. **a** Quantitative analysis of western blot analysis. Results represent the mean ± SD (*n* ≥ 3, *p < 0.05). **b** Representative result of Western Blot analysis of p-RelA

The disintegration of the NFκB:IκBα complex is induced by the phosphorylation of IκBα mediated by IKKβ, which also phosphorylates NFκB subunit RelA. Therefore, we measured both phosphorylation of IκBα on Ser32 and phosphorylation of RelA at Ser536. Pre-incubation with 240 µg/ml EA 575^®^ for 8 h was able to impede TNFα-induced phosphorylation of IκBα on Ser32 significantly (Fig. 4). Phosphorylation of RelA at Ser536 was increased after pre-incubation with 240 µg/ml EA 575^®^ and stimulation with TNFα. A significant difference in RelA phosphorylation between ivy pre-treated and control cells could be seen after 10 min of TNFα incubation (Fig. 5).

### Protein fragment complementation assay

**Fig. 6:**
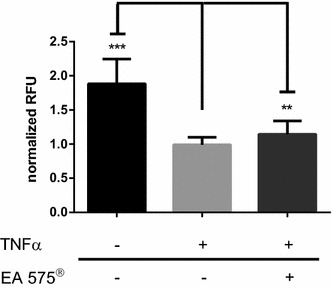
Fluorescence intensity measurement of HEK 293 cells stably transfected with cVenus-RelA and IκBα-nVenus before and after stimulation with 10 ng/ml TNFα and with or without pre-treatment using 160 µg/ml EA 575^®^ for 8 h. Results represent the mean ± SD (*n* ≥ 23, **p < 0.01, ***p < 0.001)

Reduced IκBα phosphorylation is thought to be a stabilizing factor for the NFκB:IκBα complex. Therefore, we tested via a protein fragment complementation assay if pre-incubation with EA 575^®^ enhances the stability of NFκB:IκBα under TNFα stimulating conditions. In this assay, binding of IκBα to NFκB results in the complementation of the fluorescent protein Venus. Disintegration of the complex in turn leads to a reduction of the detected Venus fluorescence (Yu et al. 2003). As shown in Fig. 6, fluorescence intensity decreased by 47.4% after stimulation with 10 ng/ml TNFα for 3 h. After pre-incubation with 160 µg/ml EA 575^®^ for 8 h, TNFα-induced fluorescence intensity was lowered to 60.8%, which represents 8.2% of conserved complex.

### Effects of EA 575^®^ on different cell types

**Fig. 7:**
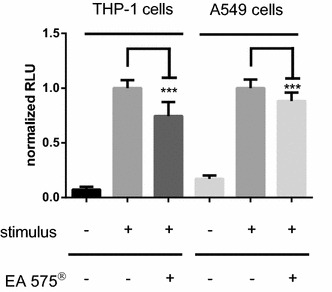
NFκB activity in THP-1 and A549 Nano-PEST cells after stimulation with 10 ng/ml LPS or 5 ng/ml TNFα, respectively, and with or without pre-treatment using 240 µg/ml EA 575^®^. Data are shown as mean ± SD of three independent experiments (*n* ≥ 16, ***p < 0.001)

To further evaluate the observed regulatory effect of EA 575^®^, we tested THP-1 as a human monocytic cell line, as well as A549 cells derived from human lung epithelium. Cells were stably transfected with the Nanoluciferase-based NFκB reporter. As can be seen in Fig. 7, both cell types showed increased Nanoluciferase levels after application of TNFα, which is significantly reduced when cells were pre-incubated with 240 µg/ml EA 575^®^. Compared to cells incubated with TNFα only, inflammatory response was reduced to 75 and 88% for THP-1 and A549 cells, respectively.

## Discussion

Ivy leaves dry extract EA 575^®^ is well established for the improvement of symptoms associated with chronic inflammatory bronchial diseases, as well as for the treatment of acute inflammatory respiratory tract infections accompanied by coughing. Molecular mechanisms behind this anti-inflammatory activity of EA 575^®^ have not yet been investigated satisfactorily.

EA 575^®^ reduces the amount of secreted IL-6 from mouse macrophages (Schulte-Michels et al. 2016a). As can be derived from our work, this reduction is achieved via an impaired nuclear translocation of NFκB, which we displayed by an immunofluorescence assay against RelA, a subunit of NFκB. Inhibition of NFκB nuclear translocation by ivy leaves constituents, e.g., rutin as well as chlorogenic and dicaffeoylquinic acids, was already shown in mast cells and macrophages (Kim et al. 2008; Puangpraphant et al. 2011; Hwang et al. 2014). Using an NFκB reporter gene assay, we could show that EA 575^®^ inhibits dose-dependently the transcriptional activity of NFκB. Decreased NFκB activity after administration of ivy constituents was already shown. For example, rutin as well as 3,5- and 4,5-dicaffeoylquinic acid inhibits the NFκB-dependent iNOS gene transcription, and as a result the synthesis of nitric oxide, whereas chlorogenic acid reduces LPS-induced NFκB-dependent cyclooxygenase-2 (COX-2) expression in RAW 264.7 cells (Shan et al. 2009; Park et al. 2009; Kazłowska et al. 2010). Furthermore, 3,4-dicaffeoylquinic acid inhibits the phorbol-12-myristate-13-acetate-induced COX-2 expression in RAW264.7 cells by blocking the catalytic activity of JNK/p38 MAP kinases and the activation of C/EBPβ and AP-1 (Han et al. 2010). The impairment of nuclear translocation and reduction in transcriptional activity of NFκB might be caused by an enhanced stability of the NFκB:IκBα protein complex. To test this issue, we performed a protein fragment complementation assay where Venus protein fluorescence is achieved upon binding of IκBα to NFκB. After stimulation with 10 ng/ml TNFα, preserved Venus fluorescence was significantly higher when cells were pre-incubated with EA 575^®^, thereby demonstrating an enhanced stability under TNFα stimulation. Complex stability is regulated by phosphorylation of IκBα through IKKβ, which forms together with IKKα and their regulating subunit IKKγ (also called NEMO) the IKK complex. Phosphorylation of IκBα on serine at position 32 (Ser32) leads to its ubiquitination and proteasomal degradation and thereby the release of NFκB from the complex, permitting it to translocate into the nucleus (Scheidereit 2006). Thus, we were interested in whether the observed effects were due to reduced phosphorylation of IκBα. A Western blot against IκBα phosphorylated at Ser32 indicated a reduced phosphorylation at this site, which explains stabilization of the NFκB:IκBα complex as shown by protein fragment complementation (see above). Reduced cellular phosphorylation of IκBα after incubation with natural products also existing in EA 575^®^ was already demonstrated, as for chlorogenic acid on hepatic stellate cells (Shi et al. 2013). Besides IκBα, IKKβ also phosphorylates the NFκB subunit RelA at Ser536. Indeed, we also found an impact on RelA-Ser536 phosphorylation which was increased after pre-incubation of cells with EA 575^®^. The phosphorylation of RelA is much less understood. Besides several phosphorylation sites which seem to have positive effects on RelA transcriptional activity, there is increasing evidence that the phosphorylation of Ser536 by IKKβ impairs the nuclear translocation of RelA and thereby diminishes its transcriptional activity (Mattioli et al. 2004; Moreno et al. 2010; Christian et al. 2016). Schröfelbauer et al. showed that the phosphorylation of IκBα on Ser32 depends on the scaffold function of NEMO and that inhibition of NEMO leads to a switch in its specificity towards RelA at Ser536, which is in good accordance with our observations (Schröfelbauer et al. 2012).

In addition to factors secreted by immune cells, those released by epithelial cells also promote inflammation of lung tissue (Mitchell and O’Byrne 2016, 2017; Green and Turner 2017). Consistent with that, the anti-inflammatory effect of EA 575^®^, detected in monocytes/macrophages (THP-1/J774.2) and examined here in detail in HEK cells, can also be observed in A 549 cells, a human lung epithelial cell. The precise target cells of EA 575^®^ in the inflamed lung should be the subject of future studies.

Taken together, our results indicate that the previously shown anti-inflammatory properties of EA 575^®^ (Schulte-Michels et al. 2016a) result from a regulatory effect on the NFκB pathway, which is probably due to a specificity switch of IKKβ. As discussed above, this would reduce RelA transcriptional activity in a duplexed manner, firstly by stabilizing the complex with its inhibitor and secondly by reducing RelA translocation into the nucleus. Whether EA 575^®^ achieves this by manipulating the scaffold function of the regulatory NEMO subunit, leading to stronger association of IKKβ to RelA compared to IκBα, must be subject of further investigations.
